# Multi-level analysis of intimate partner violence and its determinants among reproductive age group women in Ethiopia: evidence from Ethiopian Demographic Health Survey, 2016

**DOI:** 10.1186/s12889-024-18781-7

**Published:** 2024-05-23

**Authors:** Teshale Mulatu, Yadeta Dessie, Muluemebet Abera

**Affiliations:** 1 School of Nursing and Midwifery, College of Health and Medical Sciences, Haramaya University, Harar, Ethiopia; 2 School of Public health, College of Health and Medical Sciences, Haramaya University, Harar, Ethiopia; 3 Department of Population and Family Health, institute of health, Jimma University, Jimma, Ethiopia

**Keywords:** Determinants, Violence against women, Domestic violence, Intimate Partner violence, Ethiopia

## Abstract

**Background:**

Intimate partner violence (IPV) is recognized as a main public health challenge, with serious consequences for women’s physical, mental, sexual, and reproductive health. Despite its public health importance, most studies of IPV in Ethiopia mainly focused on individual characteristics and didn’t identify how factors operating at different levels affect IPV. Thus, there is limited evidence regarding the hierarchical-level factors of IPV and the effect of individual and community-level determinants of IPV. The aim of this study is to assess the individual and community-level factors associated with violence against women among ever-married reproductive-age women in Ethiopia.

**Methods:**

A retrospective analysis of secondary data retrieved from the Ethiopia Demographic and Health Survey was conducted among reproductive age group women (15–49 years of age) who reported ever being married within the available data set for the domestic violence module. STATA 14 was used to conduct the analysis. A two-level mixed-effects logistic regression analysis was used to determine associations between IPV and individual- and community-level factors. IPV variability across the community was assessed using ICC and PCV. The model’s fitness was assessed using the Akaike information criterion (AIC), the Bayesian information criterion (BIC), and the likelihood ratio test.

**Result:**

The life time prevalence of IPV in this study was 33% [95% CI: 30.74, 34.25]. Women’s age 20–24 (AOR = 5.85, 95% CI: 201 3.10, 11.04), 25–29 age group (AOR = 6.41, 95% CI; 3.34, 12.32), 30–34 age group (AOR = 9.48, 95% CI: 4.71, 19.06), 35–39 age group (AOR = 9.88, 95% CI: 4.79, 20.39), 40–44 age group (AOR = 11.10, 95% CI: 5.16, 23.89), and 45–49, (AOR = 14.15, 95% CI: 6.01, 32.80), early marriage (AOR = 1.21, 95% CI: 1.08, 1.47), witnessing inter-parental violence during childhood (AOR = 2.80, 95% CI: 2.16, 3.96), having a lot of living children (AOR = 0.45, 95% CI: 0.26, 0.74), having a partner who drank alcohol (AOR = 3.00, 95% CI: 2.42–3.67), decision-making autonomy of the women (AOR = 0.77, 95% CI: 0.62, 0.97), Poor wealth index (AOR = 1.64, 95% CI: 1.23, 2.18), middle wealth index (AOR = 1.86, 95% CI: 1.36, 2.54) and exposure to media (AOR = 1.47, 95% CI: 1.06, 2.00) were all significantly associated with IPV.

**Conclusion and recommendation:**

This study showed that one-third of the women experienced IPV in their lifetime. The finding suggested that community based interventions and multi-sectorial collaborations are needed to reduce the IPV and its adverse consequences.

## Background

Intimate partner violence (IPV) is a serious global public health concern and a blatant violation of women’s human rights. It denotes any act or pattern of behavior (aggression, coercion and abuse) by an intimate partner or ex-partner that cause physical, sexual, psychological and emotional harm to the spouse or who are with in close relationship [1].

The World Health Organization (WHO) estimates the global prevalence of IPV to be 30–35% [2, 3]. Lifetime intimate partner violence prevalence (ever experience of at least one form of IPV throughout reproductive life) estimates range from 20% in the Western Pacific, 22% in high-income countries and Europe, and 25% in the WHO Americas Regions to 33% in the WHO African region, 31% in the WHO Eastern Mediterranean region, and 33% in the WHO South-East Asia region [3, 4].

Surveys in Ethiopia have also revealed that greater than one-third(35%) of ever-married women report that they have experienced physical, emotional, or sexual violence from their husband or partner at some point in time [5].

Women who experience intimate partner abuse suffer severe short- and long-term physical, mental, sexual, and reproductive health problems. It results in injuries, unintended pregnancies, induced abortions, gynecological disorders, and STDs, including HIV [6].

About 38% of all murders of women are committed by intimate partners worldwide, and a staggering 42% of women who have encountered it suffered injuries as a result of the abuse [2, 4]. Abuse by an intimate partner during pregnancy also raises the risk of miscarriage, stillbirth, premature birth, and low birth weight [7–10]. Intimate partner abuse may result in depression, post-traumatic stress disorder, and other anxiety disorders, sleeping difficulties, eating problems, and suicidal ideation [11, 12].

For women, their families, and societies at large, this violence has serious social and economic consequences. As a result of intimate partners and sexual abuse, women face isolation, an unwillingness to work, a lack of interest in daily activities, and a failure to take care of themselves and their families [13, 14].

The Sustainable Development Goals (SDGs) place a strong emphasis on the need to stop violence against women as a means to advance gender equality and women’s empowerment (SDG-5). To stop violence against women, the World Health Organization (WHO) and United nations (UN) have created the “RESPECT Women” platform in partnership with multi-sectorial agencies [14, 15].

With cooperation from UN-Women and UNICEF (United Nations International Children and Emengency Fund), the Ethiopian government also established a five-year plan (2021/22-2026) for the prevention and response to violence against women and children. The policy’s main goals are to strengthen reporting mechanisms, provide support services for violence survivors, and allow women and children to exercise their rights without fear of violence [16].

Although great efforts have been made to identify, combat, and prevent all forms of IPV, there is still little investment in IPV research and coordination for tracking progress towards the SDGs in most low and middle income countries, including Ethiopia [17–19].

Even though there have been studies on the factors of intimate partner violence (IPV) in Ethiopia, the majority of these studies were small-scale studies conducted in single and limited settings that lacked national representativeness. Moreover, due to hierarchical-level causal factors it is imperative to employ strong analytical techniques to precisely determine the impact of IPV determinants in particular populations. Investigation of the contributing factors for IPV at the individual and community level aids in developing preventive and controlling strategies to tackle it. This study, therefore, aimed to examine determinants of IPV at the individual and community level among ever-married reproductive-age women in Ethiopia.

## Methods and material

### Study setting and period

Ethiopia is comprised of two administrative cities (Addis Ababa city administration and Dire Dawa city council) and nine national regional states, including Tigray, Afar, Amhara, Oromia, Somali, Benishangul-Gumuz, Southern Nations Nationalities and Peoples’ Region (SNNPR), Gambella, and Harari. There are 16,253 kebeles (a country’s smallest administrative unit), 817 districts, and 68 zones in this country [5]. The EDHS 2016 study period ran from January 18, 2016, to June 27, 2016.

### Sampling procedure, study population and sample size

A two-stage stratified cluster sampling were used for the selection of 2016 EDHS sample. In the first stage, a total of 645 enumeration regions (EAs)—202 in urban regions and 443 in rural areas—were chosen independently within each sampling stratum with a probability proportional to the size of the EAs. In the second stage, an average of 28 households from each primary sampling unit was selected using systematic random sampling. A total of, 15,683 women who were 15–49 years of age and who reported ever being married participated in the survey for the domestic violence module. Only one married woman per household was selected and those who completed the IPV questionnaire were included in the analysis. The sample was weighted to adjust for unequal probabilities of selection that might have occurred during sampling and to enhance the representativeness of results for generalization/inference (weighted sample = 2,734).

### Study variables and measurements

#### Independent variables

Both individuals’ socio-demographic characteristics and relationship-related variables were considered as level one variable because in the EDHS only one woman per household was sampled. Hence household- or relationship-related factors were included in the level one variable (collectively labeled as individual level variables). Hence household- or relationship-level clustering may not exist.

##### Individual level variables

The women’s characteristics included age at first cohabitation, educational status, attitudes towards wife-beating and intra-parental violence witnessed during childhood. The partner-related factors examined were education, age, and alcohol consumption. Relationship related factors included women’s decision-making autonomy, wealth index, and number of living children.

**Community-level variables** included residency and media exposure.

##### Dependent variable

self-reported life time partner violence.

### Measurement

In accordance with the 2016 EDHS, women were considered to have lifetime IPV if they stated that they were ever victimized for at least one instance of physical, emotional, or sexual violence since the age of 15 throughout their reproductive age [5].

#### Physical violence

push you, shake you, or throw something at you; slap you; twist your arm or pull your hair; punch you with his or her fist or with something that could hurt you; kick you, drag you, or beat you up; try to choke you or burn you on purpose; or threaten or attack you with a knife, gun, or any other weapon. The respondents were categorized as having experienced lifetime physical violence if they reported at least one of those listed acts since the age of 15 [5].

#### Emotional violence

say or do something to humiliate you in front of others; threaten to hurt or harm you or someone close to you; insult you or make you feel bad about yourself. The respondents were categorized as having experienced lifetime emotional spousal violence if they reported at least one of those listed acts since the age of 15 [5].

#### Sexual violence

physically force you to have sexual intercourse with him even when you did not want to; physically force you to perform any other sexual acts you did not want to; force you with threats or in any other way to perform sexual acts you did not want to. The respondents were categorized as having experienced lifetime sexual violence if they reported at least one of those listed acts since the age of 15 [5].

**Women’s attitudes towards wife beating** were measured based on the following five questions: whether situations of hitting or beating a wife are justifiable: if she goes out without telling him; neglects their children; argues with him; refuses to have sex with him; and burns the food. If they said ‘yes’ to any one of the above questions, they were classified as having an attitude towards beating their wives.

**Women’s decision-making autonomy** was categorized as ‘yes’ if a woman was involved in all decisions regarding her income, own health care, major household purchases, and visits to family or relatives.

**Witnesses to inter-parental violence** were assessed by ‘yes’ or ‘no’ based on their answer to the question, “As far as you know, did your father ever hit your mother?”

#### Data management and analysis

STATA 14 was used to conduct the analysis. Prior to conducting any statistical analysis, the data were weighted using the sampling weight (weight for domestic violence), primary sampling unit, and stratum to restore the survey’s representativeness, taking the sampling design and standard errors into account to provide accurate statistical estimations. Text and tables were used to display descriptive statistics and summary statistics.

The correlations between IPV and individual- and community-level characteristics were examined using a two-level mixed-effects logistic regression analysis. This model was used in order to prevent the clustering effects of factors acting at various levels on the outcome variable.

Four models were fitted using mixed effect logistic regression analysis. The subsequent models were constructed by adding covariates at each level on the preceding model. The measures of association (fixed effects) were presented using adjusted odds ratio together with 95% CI (AOR at 95% CI). The variable was deemed significant if its *p*-value was less than 0.05.

The primary model (null or empty) was fitted without explanatory variables. The second model (individual/relationship level), third model (community level), and fourth model (individual and community level combined) variables were fitted accordingly. The final model was used to test whether the community-level and individual-level variables had an independent impact on the IPV experience. The intra-cluster correlation (ICC) was used to show cluster correlation within a model. The predictive ability of the variables used in each model was also measured using the proportional change in variance (PCV).

The Akaike information criterion (AIC), Bayesian information criterion (BIC) and likelihood ratio test were used to evaluate the model’s fitness. A comparison was made between the AIC and BIC values for each model, with the lowest value presumed to be the best explanation. The variance inflation factor (VIF) was used to examine the possibility of multicollinearity between the variables at the individual and community levels.

## Results

### Characteristics of the study participants

A total of 2,734 ever married women in the reproductive age group were included in the analysis. One-fourth (25.09%) of study participants were between the ages of 25 and 29. About 36.05% of the women were married before turning 18 years, 62.43% of them had no formal education, and 68.87% were unemployed.

Regarding intimate partners, the majority of them were literate (54.6%) and younger than 35 years old (58.53%). About 70% of participants stated that their husbands or partners drank alcohol, while the majority of participants (69.14%) reported having decision-making autonomy. Regarding community-level characteristics, the majority of respondents (85.5%) lived in a rural area and had no media exposure (78.59%) (Table 1).

**Table 1:** Characteristics of study participants among ever married women in Ethiopia, *n* = 2734

Variables	Category	Frequency	Percentage
Age	15–19	137	5.02
	20–24	453	16.56
	25–29	686	25.09
	30–34	577	21.11
	35–39	464	16.97
	40–44	283	10.36
	45–49	134	4.88
Religion	Orthodox	1,032	37.8
	Muslim	1,107	40.5
	Protestant	501	18.3
	*Others	94	3.4
Residence	Urban	387	14.15
	Rural	2,347	85.85
Age at first cohabitation	< 18	985	36.05
	≥ 18	1749	63.95
Maternal education status	No formal education	1,707	62.43
	Primary	762	27.86
	Secondary and above	265	9.70
Maternal working status	Unemployed	1,883	68.87
Maternal working status	Unemployed	1,883	68.87
	Employed	851	31.13
Age of partner	< 25	135	4.94
	25–34	999	36.53
	≥ 34	1,600	58.53
Partner education			
	Illiterate	1,240	45.35
	Literate	1,494	54.65
House hold wealth index	Poor	1,067	39.05
	Middle	574	20.98
	Rich	1,093	39.97
Exposure to media	No	2,149	78.59
	Yes	585	21.41
No. of living children	None	205	7.50
	1–2	822	30.05
	3–4	805	29.46
	≥ 5	902	32.99
Decision making autonomy	No	844	30.86
	Yes	|1,890	69.14
Justify wife beating	No	867	31.72
	Yes	1,867	68.28
Witness intra parental violence	No	1,995	72.99
	Yes	739	27.01
Partner drinks alcohol	No	1,908	69.77
	Yes	826	30.23

### Prevalence of intimate partner violence

About one-third (33%)[95% CI: 30.74, 34.25] of the women encountered IPV. Physical (22.45%) [95% CI: 20.93, 24.06] and emotional (22.46%) [95% CI: 20.94, 24.07] violence were the most commonly reported types of IPV, whereas sexual violence was the least reported form of IPV (9.64%) [95% CI: 8.59, 10.81].

### Determinants of IPV

According to random effects analysis, model I had no individual-level or community-level variables, and it observed only the random intercept variables. The ICC in the null model (model 1) showed that the IPV experience of women within the community has a higher clustering variation (ICC = 23.4%).

Also, cluster variability (ICC) in final model (combined individual-level and community level factors) indicates that 26.3% of IPV was explained by cluster differences in the community.

Similarly, the PCV in the final model (combined individual-level and community-level factors) indicates that 16.17% of variation in IPV was explained by the combined effect of the individual and community-level factors.

In the bi-variable analysis, maternal age, age at first cohabitation, maternal education, husband education, residence, wealth index, exposure to media, witnessing inter-parental violence during childhood, having a lot of living children, having a partner who drank alcohol, justifying wife beating acceptable and women’s decision-making autonomy were significantly associated with IPV(Table 2).

**Table 2:** Experience of IPV by characteristics of study participants, among women in Ethiopia, EDHS 2016, (*n* = 2734)

		IPV		*P*-Values
Variables	Categories	YES (%)	NO (%)	
Age	15–19	116 (83.5)	23(16.5)	< 0.001***
	20–24	306(67.5)	147(32.5)	
	25–29	|468(68.2)	218(31.8)	
	30–34	375(64.9)	202(35.1)	
	35–39	304(65.7)	159(34.3)	
	40–44	188(66.4)	95(33.6)	
	45–49	90(67.7)	43(32.3)	
Age at first cohabitation	< 18	1,145(65.5)	603(34.5)	0.009**
	≥ 18	701(71.1)	285(28.9)	
Maternal education status	No formal education	1,105(64.7)	602(35.2)	< 0.001 ***
	Primary	524(68.8)	238(31.2	
	Secondary and above	217(81.9)	48(18.1)	
Age of partner	< 25	101(74.8)	34(25.2)	0.144
	25–34	662(66.2)	337(33.7)	
	≥ 35	1083(67.7)	517(32.3)	
Husband education	Illiterate	789(64.1)	442(35.9)	0.031*
	Literate	1048(70.1)	446(29.9)	
Wealth index	Poor	681(63.8)	386(36.2)	<0.001***
	Middle	355(61.8)	219(38.2)	
	Rich	809(74)	284 [20]	
Number of living children	None	152(73.8)	54(26.2)	0.027*
	1–2	561(68.3)	260(31.7)	
	3–4	562(69.8)	243(30.2)	
	≥ 5	571(63.3)	331(36.7)	
Decision making autonomy	No	520(61.7)	323(38.3)	0.003**
	Yes	1326(70.1)	565(29.9)	
Witness interparental violence	No	1,452(72.8)	543(27.2)	< 0.001***
	Yes	394(53.3)	345(46.7)	
Justify wife beating	No	625(72.1)	242(27.9)	0.01**
	Yes	1221(65.4)	646(34.6)	
Partner drinks alcohol	No	1,354(71)	553 [21]	<0.001**
	Yes	492(59.5)	335(40.5)	
Type of place residence	Rural	1,540(65.6)	807(34.4)	0.005**
	Urban	306(79.1)	81(20.9)	
Exposure to media	No	1,393(64.9)	755(35.1)	<0.001***
	Yes	453(77.3)	133(22.7)	

Significant at *P-value < 0.05, **P-value < 0.01, ***P-value < 0.001

After adjusting for both individual and community-level factors in the final model, women’s age, early marriage, number of living children, having experienced inter-parental violence as a kid, having a partner who drank alcohol, autonomy in decision-making, income index, and exposure to social media were all strongly associated with IPV (Table 3).

**Table 3:** Multivariable logistic regression analysis of individual, community, and individual-community level factors associated with intimate partner violence among women in Ethiopia, EDHS 2016, (*n* = 2734)

		Model I Null model	Model II Individual level	Model III Community level	Model IV Individual-community level	
**Variables** **Age**	**Category**		**AOR (95%CI)**	**AOR (95% CI)**	**AOR (95% CI)**	***P***-**value**
	15–19		1		1	
	20–24		5.79(3.07,10.89) ***		5.85(3.10, 11.04) ***	
	25–29		6.28(3.28, 12.02) ***		6.41(3.34, 12.32) ***	<0.001
	30–34		9.17(4.58, 18.33) ***		9.48(4.71, 19.06) ***	
	35–39		9.73(4.73, 19.94) ***		9.88 (4.79, 20.39) ***	
	40–44		11.01(5.14,23.56) ***		11.10(5.16, 23.89) ***	
	45–49		14.14(6.11,32.55) ***		14.15(6.01, 32.80) ***	
**Age at first cohabitation**	< 18		1.40(1.10, 1.76) **		1.40 (1.10, 1.77) **	0.005
	≥ 18		1		1	
**Educational status**	No formal education		1		1	
	Primary		0.92(0.72, 1.21)		0.95(0.73, 1.25) 0.56	
	Secondary and above		0.57(0.36, 0.93)		0.65(0.39, 1.10) 0.09	
**Age of the partner**	< 25		1		1	
	25–34		1.15(0.67, 1.98)		1.16(0.67, 1.99) 0. 66	
	≥ 35		0.63(0.35, 1.15)		0.63(0.34, 1.14) 0.13	
**Partner education**	Illiterate		1		1	
	Literate		1.02(0.81, 1.29)		1.04(0.83, 1.32) 0.604	
**Wealth index**	Poor		1.77(1.34, 2.33) ***		1.64 (1.23, 2.18) ** 0.001	
	Middle		2.00 (1.48, 2.71) ***		1.86(1.36, 2.54) ** 0.001	
	Rich		1		1	
	None 1–2		1 0.87(0.54, 1.35)		1 0.86(0.54, 1.37) 0.520	
**Number of living children**	3–4 > 4		0.46(0.27, 0 0.81) * 0.45(0.26, 0.74) **		0.48(0.264, 0.81) * 0.48(0.264, 0.81) *	0.03 0.012
**Decision making autonomy**	No		1		1	
	Yes		0.76(0.61, 0.96) **		0.77(0 0.62, 0.97) *	0.028
**Witness interparental violence**	No		1		1	<0.001
	Yes		3.09(2.46, 3.88) ***		3.12(2.48, 3.92) ***	
**Justify wife beating**	No		1		1	
	Yes		1.22(0.97, 1.54)		1.20(0.95, 1.52)	0.12
**Partner drinks** **alcohol**	No		1		1	
	Yes		2.34(1.82, 3.01) ***		2.39 (1.86, 3.08) ***	<0.001
**Residence**	Rural			1.29(0.84, 1.97)	1.00 (0.60, 1.66)	0.99
	Urban			1	1	
**Exposure to media**	No			1.67(1.25,2.22)**	1.47 (1.06, 2.04) *	0.021
	Yes			1	1	
**Random effect**		**Model I**	**Model II**	**Model III**	**Model IV**	
Community level variance (SE)		1.1(0.16) *	1.17(0.18) *	0.96(0.15) *	1.17 (0.19) *	
ICC%		23.4	26.3	22.6	26. 25	
MOR		2.61	2.81	2.55	2.81	
PCV %		Reference	16.52	4.82	16.17	
**Model fit statistics**						
Log-likelihood		− 1618.93	-1489.54	-1608.83	-1486.71	
AIC		3175.76	3025.09	3225.66	3023.43	
BIC		3187.56	3160.71	3249.25	3170.84	

Significant at *P-value < 0.05, **P-value < 0.01, ***P-value < 0.001, 1 = reference

AOR = Adjusted Odds Ratio; CI = Confidence Interval; SE = Standard Error;

ICC = Intra-class Correlation Coefficient; PCV = Proportional Change in Variance; AIC = Akaike Information Criterion; BIC = Bayesian information criterion

Model I is the empty model or a baseline model without any determinant variables

Model II is adjusted for individual-level factors

Model III is adjusted for community-level factors

Model IV is the final model adjusted for individual and community -level factors

This study showed that the odds of IPV increase among older women compared to the younger age groups. Those women whose ages were 20–24 were about six times (AOR = 5.85, 95% CI: 3.10, 11.04) more likely to experience IPV compared to those whose ages were 15–19. Similarly, the odds of IPV were six times 6.41 (3.34, 12.32), nine times 9.48 (4.71, 19.06), ten times 9.88 (4.79, 20.39), eleven times 11.10 (5.16, 23.89), and fourteen times 14.15 (6.01, 32.80) higher among age groups of 25–29, 30–34, 35–39, 40–44, and 45–49, respectively. On the contrary, women aged *<* 18 years at first marriage had higher odds of IPV (AOR = 1.21, 95% CI: 1.08, 1.47) compared to women aged ≥ 18 years at first marriage.

Women who had witnessed inter-parental violence during childhood were about three times **(**AOR = 2.80, 95% CI: 2.16, 3.96**)** more likely to report IPV compared to women who had not witnessed inter-parental violence during childhood. Women who had decision-making autonomy in the household were 33% less likely (AOR = 0.77, 95% CI: 0.62, 0.97) to experience IPV compared to women who had no decision-making autonomy. Those women who had 3–4 children were 55% less likely [AOR = 0.45 (95% CI: 0.26, 0.74)] and those who had ≥ 5 children were 52% less likely [AOR = 0.48 (95% CI: 0.264, 0.81)] to experience IPV compared to those women who had no living children.

Regarding partner’s behavior, women whose partner drank alcohol were three times (AOR = 3.00, 95% CI: 2.42–3.67) more likely to experience IPV compared to those women whose partner did not drink alcohol. Exposure to media is another important predictor of IPV at the community level. Those women who had no exposure to any form of media at least once in a week were 1.5 times [AOR = 1.47, 95% CI: 1.06, 2.04] more likely to encounter IPV compared to those who had exposure to media. In addition, women with poor wealth index were 1.6 times (AOR= 1.64, 95% CI: 1.23, 2.18), and those with middle wealth index were about 1.9 times (AOR=1.86, 95% CI: 1.36, 2.54) more likely to encounter IPV compared to the richer. 

## Discussion

Ending violence against women, especially intimate partner violence (IPV), is one of the vital strategies to achieve the Sustainable Development Goals (SDGs), which encompass gender equality and women’s empowerment. Hence, this study attempted to examine the magnitude of IPV and its determinants using the national demographic survey data.

This study showed, that one-third (33%), [95% CI: 30.74, 34.25] of the women have experienced IPV in their lifetime. Women’s age, early marriage, number of living children, witnessing inter-parental violence during childhood, having a partner who drank alcohol, decision-making autonomy, wealth index, and exposure to social media were significantly associated with IPV.

The magnitude of IPV in this study was in line with the national survey report (35%) [5], WHO estimates for IPV (30%) [1], and the overall prevalence of IPV in East African countries (32.66%) [22]. The prevalence of IPV in this study is higher than that of Nigeria (15.2%) [23]. However, it is lower than other studies conducted in Uganda (56%) [24], Tanzania (46%) [25], Gambia (39.23%) [26], and Liberia (44.74%) [27]. The possible explanation for the observed differences might be due to the difference in the study population, the sample size they used, cultural taboos, and the implementation of laws that prevent IPV.

This study showed that the odds of IPV increase among older women. This finding was congruent with other study findings [20, 26–28].The explanation could be that older women are more likely to report their lifetime cumulative exposure to IPV than younger women. As the age advances the time of marriage also increases so partner may commit violence on one occasion due to different reasons.

On the contrary, women aged *<* 18 years at first marriage had higher odds of IPV compared to women aged ≥ 18 years at first marriage. This may be because women who married at an early age were not mature enough to protect themselves from harm and psychological infliction from their partners, which might increase the risk of IPV. This finding was in line with evidence from other studies [21, 27, 29, 30].

Women who witnessed inter-parental violence during their childhood were 2.8 times more likely to experience IPV. This finding might be explained by a phenomenon in which young girls who experience violence grow up to accept it as normal and have a normative perspective on it; as a result, they are more likely to bear assault from their spouses and have a passive reaction. This is supported by other study findings [20, 27, 28].

In this study, women whose partner drank alcohol had increased odds of IPV. This is similar with previous research findings (31–34). This might be due to the strong influence of alcohol on behavior. Excessive alcohol drinking may affect the cognitive function of the mind, reducing self-control and makes individuals incapable of a peaceful resolution to conflicts.

Women’s decision-making autonomy in a relationship was found to be a protective factor against IPV. Women with decision-making autonomy in the household were 33 % less likely to report experience of IPV compared to women who had no decision-making autonomy. The possible reason might be women who have a decision-making power are empowered to decide on important issues and may protect their right. This study finding is backed by the findings reported in other studies [27, 31, 32, 33, 34].

This study also showed that the household wealth status is significantly associated with IPV. Women with poor and middle wealth status were more likely to be exposed to IPV compared to those women with rich wealth status. Although violence occurs in all socioeconomic groups, it is more frequent and severe in lower groups and developing nations. This finding is supported with other study findings [34–37].

This study revealed that having a lot of living children is a protective factor for IPV. Those women who had ≥ 5 children were 52% less likely to experience IPV compared to those women who had no living children. This finding was contrary to other study findings, which reported a higher number of children or family size as a risk factor for IPV [37–39]. Children can serve as a protective factor by creating a sense of purpose and responsibility for parents. The responsibility of caring for multiple children may motivate women to seek help or find ways to protect themselves and their children from abuse. Another possible justification could be in some cultures, having a large family is considered desirable and socially valued. Women with many children may receive more social recognition and respect, which can indirectly reduce their vulnerability to IPV.

Exposure to media is another important predictor of IPV at the community level. Those women who had no exposure to any form of media at least once in a week were 1.5 times more likely to encounter IPV compared to those who had exposure to media. This might be due to the influence of mass media on women’s attitudes regarding reproductive health rights and women’s empowerment to prevent violence against women. This finding is supported by other study findings [40, 41].

This study has implications for policymakers and programmers as it is based on a solid statistical analysis and the most representative national data. The evidence can be taken into account when designing future IPV prevention programs that aim to improve factors that operates at different levels. Nevertheless the following restrictions must be taken into account when interpreting the result of this study. It is challenging to establish cause-effect linkages in the study because it is cross-sectional in nature. Even though the data are representative, there may still be under-reporting of IPV episodes because of shame, stigma, and fear of penalties. Finally, because all the factors—including partner traits—were self-reported, recall bias may have affected the estimates of IPV.

## Conclusion

The findings of this study indicated that Ethiopia has a high proportion of IPV. This high prevalence of IPV against women suggests the needs of strengthening women’s empowerment and ensuring effective national strategies for prevention of IPV. The findings of this study implied that multi-sectoral cooperation and the participation of many stakeholders from communities as well as governmental and non-governmental organizations are needed in order to stop the intergenerational cyclic effect of IPV. Future research should concentrate on qualitative projects that could investigate how social dynamics contribute to and sustain IPV in communities.

## Data Availability

All related data are presented fully within the paper. Additional data are accessible from the correspondent author upon reasonable request.

## **Abbreviations/Acronyms**

EDHS: Ethiopian Demographic Health Survey
IPV: Intimate Partner Violence
SDG: Sustainable Development Goal
STD: Sexually Transmitted Diseases
UN: United Nations
UNICEF: United Nations International Children Emergency Fund
WHO: World Health Organization
